# Hand Book of Insanity for Practitioners and Students

**Published:** 1893-05

**Authors:** 


HANDBOOK OF INSANITY FOR PRACTITIONERS AND STUDENTS—By Dr. Theodore Kirchoff, Physician to the Schleswig Insane Asylum and Privat-Docent at the University of Keil, Published by William Wood & Co., New York, 1893.

Dr. Kirchoff has written a book on insanity, that will do much good. He has not treated the subject exhaustively, as many others, yet on account of its brevity, and simplicity many will read it, when the lengthier works will remain unread. The work is in good form and well gotten up. The plates are very good, and, on the whole, is meritorious.

